# Prevalence and factors associated with overweight, obesity and central obesity among adults in Shenmu City, Shaanxi Province, China

**DOI:** 10.1016/j.pmedr.2024.102673

**Published:** 2024-03-03

**Authors:** Mingxia Liu, Chunjiao Jia, Yaoda Hu, Juan Liu, Lizhen Liu, Shengli Sun, Haiying Wang, Yonglin Liu

**Affiliations:** a Department of Prevention and Health Care, Shenmu Hospital, The Affifiliated Shenmu Hospital of Northwest University, Shenmu, China; b Medical Department, Institute of Basic Medical Sciences Chinese Academy of Medical Sciences, School of Basic Medicine Peking Union Medical College, Beijing, China; c Department of Epidemiology and Statistics, Institute of Basic Medical Sciences Chinese Academy of Medical Sciences, School of Basic Medicine Peking Union Medical College, Beijing, China; d Department of Prevention and Health Care, Institute of Basic Medical Sciences Chinese Academy of Medical Sciences, School of Basic Medicine Peking Union Medical College, Beijing, China; e Ultrasound Medicine Department, Institute of Basic Medical Sciences Chinese Academy of Medical Sciences, School of Basic Medicine Peking Union Medical College, Beijing, China; f Department of Neurology, Shenmu Hospital, The Affifiliated Shenmu Hospital of Northwest University, Shenmu, China; g Science and Education Department, Shenmu Hospital, The Affifiliated Shenmu Hospital of Northwest University, Shenmu, China

**Keywords:** Shenmu City, Overweight, Obesity, Central obesity, Prevalence, Factors

## Abstract

**Objectives:**

This research aimed to explore the prevalence and determinants of overweight, obesity, and central obesity in Shenmu City, Shaanxi Province, China and to offer guidance for preventative health measures.

**Methods:**

We conducted a multi-stage, stratified random sampling survey among 4,565 residents of Shenmu City. Data collection included questionnaires and anthropometric assessments to gather socio-demographic data and to identify cases of overweight, obesity, and central obesity. Multivariable logistic regression analysis was utilized to assess the association between various factors and these conditions.

**Results:**

The observed prevalence rates for overweight, obesity, central obesity, and the combination of overweight/obesity with central obesity were 39.9%, 18.2%, 48.0%, 32.8%, and 22.8%, respectively. Notably, the incidence of these conditions was significantly higher in men compared to women. The prevalence of overweight and obesity initially increased and then decreased with age, whereas the prevalence of central obesity consistently rose. Furthermore, a higher educational level correlated with lower prevalence rates. Additionally, our analysis indicated that hypertension, dyslipidemia, and hyperuricemia are risk factors for these conditions.

**Conclusions:**

The findings of this study offer crucial insights for formulating effective strategies to prevent and manage obesity in Shenmu City.

## 1 Introduction

Overweight, obesity, and central obesity are characterized as chronic metabolic disorders stemming from excessive total fat accumulation and/or localized fat increase with abnormal distribution. These conditions are commonly influenced by a synergy of genetic and environmental factors (Wei et al., 2022, Panuganti et al., 2022). In recent years, the prevalence of these conditions has escalated into a global public health concern due to economic advancement and improved living standards (Apovian et al., 2022, Pan et al., 2021, Hu et al., 2017). In 2016, it was reported that over 1.9 billion adults worldwide, aged 18 and older, were overweight, with more than 650 million classified as obese. The Report on Nutrition and Chronic Diseases in China (2020) indicates that 34.3 % of Chinese adults are overweight, and 16.4 % are obese, highlighting the significant public health challenge these conditions pose both globally and within China.

The impact of overweight, obesity, and central obesity extends beyond physical health, affecting mental well-being significantly. Research indicates a higher propensity for mental health issues, particularly anxiety and depression, among those affected by these conditions (Fulton et al., 2022). Moreover, overweight and obesity are not only chronic conditions in their own right but are also associated with a myriad of other chronic diseases including diabetes, stroke, coronary artery disease, hypertension, respiratory diseases, obstructive sleep apnea, osteoarthritis, and gallstones (Zhang et al., 2013, Powell-Wiley et al., 2021, Ibrahim and El-Sayed, 2021, Parra-Landazury et al., 2021). Furthermore, there is a well-documented link between obesity and an increased risk of various cancers, such as breast, colorectal, liver, among others (Larsson and Burgess, 2021, Maguire et al., 2021, Lee et al., 2019, Ma et al., 2013, Parida et al., 2019). Notably, studies have also identified obesity as a significant independent risk factor for adverse outcomes, including mortality, in cases of novel coronavirus pneumonia, with the severity of outcomes correlating with the degree of obesity (Simonnet et al., 2020, Zhou et al., 2021).

While the prevalence of overweight, obesity, and central obesity is widespread and affects all populations, significant geographical disparities exist. Despite numerous studies, there is a lack of large-scale data on the prevalence of these conditions in Shenmu City, Shaanxi Province. Thus, our study aims to assess the relationship between various risk factors and the prevalence of overweight, obesity, and central obesity among 4,565 adult residents of Shenmu City. The findings are expected to guide the development of health living guidelines for the local population and provide theoretical support for crafting relevant public health prevention policies.

## 2 Materials and methods

### 2.1 Study participants

A multi-stage, stratified random sampling methodology was employed in Shenmu City, Shaanxi Province, China. Between August and December 2019, we selected adult participants from two communities (Linzhou and Yingbin Road) and four towns (Jinjie, Daliuta, Hejiachuan, Banbanbao) within Shenmu City for our survey. Initially, data from a total of 4,706 individuals were collected. After excluding 141 participants due to incomplete information, the final sample size comprised 4,565 individuals. This study received ethical approval from the Ethics Committee of Shenmu Hospital (Approval No. sm004), and informed consent was obtained from all participants.

### 2.2 Survey content

The survey consisted of two primary components: questionnaires and anthropometric measurements. The questionnaire captured various demographic and health-related data, including age, sex, place of residence, educational level, marital status, and medical history. Anthropometric measurements were conducted by trained medical personnel and encompassed height, weight, waist circumference (WC), and blood pressure. The Body Mass Index (BMI) was calculated using the standard formula: weight in kilograms divided by the square of height in meters.

### 2.3 Diagnostic criteria

(1) Overweight, obesity and central obesity were defined according to the Adult Weight Determination (WS/T 428–2013), 18.5 kg/m^2^ ≤ BMI < 24.0 kg/m^2^ means normal weight, 24.0 kg/m^2^ ≤ BMI < 28.0 kg/m^2^ means overweight, BMI ≥ 28.0 kg/m^2^ means obesity (Wang et al., 2014), and men waist circumference ≥ 90 cm and women waist circumference ≥ 85 cm means central obesity (Lim and Park, 2018).
(2) Hypertension: Hypertension is defined as having a systolic blood pressure of 140 mmHg or higher and/or a diastolic blood pressure of 90 mmHg or higher. This definition applies regardless of whether the individual is taking antihypertensive medication (Yin et al., 2022).
(3) Diabetes: Diabetes is defined as a fasting blood glucose level of 7.0 mmol/L or higher, or current use of hypoglycemic drugs or insulin (Jia et al., 2019).
(4) Dyslipidemia: Dyslipidemia is diagnosed when one or more of the following criteria are met: total cholesterol (TC) levels of 5.2 mmol/L or higher, triglycerides (TG) levels of 1.7 mmol/L or higher, and/or low levels of high-density lipoprotein cholesterol (HDL-C), specifically less than 1.0 mmol/L (Song et al., 2020).
(5) Hyperuricemia: Hyperuricemia is defined as having a fasting blood uric acid level exceeding 420 μmol/L in adults (Li et al., 2019).“

### 2.4 Statistical analyses

SPSS 25.0 software was used for descriptive statistics of all variables, *t*-test for continuous variables and chi-square test for categorical variables. Count data were expressed as the number of cases (%). χ^2^ test and multivariable logistic regression were used to analyze the prevalence and epidemic characteristics of overweight, obesity and central obesity. A significance level of less than 0.05 (*p* < 0.05) was considered statistically significant.

## 3 Results

### 3.1 Socio-demographic characteristics

A total of 4,565 adult residents of Shenmu City participated in this study, comprising 1,826 men and 2,739 women. The socio-demographic characteristics of the participants are detailed in Table 1. Men generally exhibited higher average height, weight, BMI, WC, systolic and diastolic blood pressure, and age compared to women, with these differences being statistically significant (*p* < 0.001). In terms of weight categories and central obesity, the incidence rates of overweight, obesity, and central obesity were significantly higher in men than in women. Age-wise, the population of men over 60 was significantly larger than that of women in the same age bracket. Regarding education, women with a primary school education or less outnumbered their male counterparts, approximately doubling their number. Additionally, the prevalence of diabetes, dyslipidemia, and hyperuricemia was higher among men than women. However, no significant differences were observed in living areas, marital status, or the prevalence of hypertension between the genders.

**Table 1:** Socio-demographic characteristics of the studied adults in Shenmu city, Shaanxi province, 2019.

Characteristic	Total	Men	Women	*p*
**Total, n (%)**	4565 (100.0)	1826 (40.0)	2739 (60.0)	
Height values (cm), mean ± SD	163.07 ± 7.76	168.85 ± 6.56	159.22 ± 5.90	**< 0.001^a^**
Weight values (kg), mean ± SD	66.32 ± 11.23	72.32 ± 11.33	62.32 ± 9.21	**< 0.001^a^**
BMI values (kg/m^2^), mean ± SD	24.89 ± 3.53	25.34 ± 3.53	24.59 ± 3.51	**< 0.001^a^**
Waist Circumference (cm), mean ± SD	85.86 ± 10.26	90 ± 9.55	83.09 ± 9.78	**< 0.001^a^**
Systolic blood pressure (mmHg), mean ± SD	127.4 ± 18.76	128.99 ± 17.65	126.33 ± 19.40	**< 0.001^a^**
Diastolic blood pressure (mmHg), mean ± SD	80.15 ± 12.31	82.08 ± 12.09	78.86 ± 12.29	**< 0.001^a^**

**Weight categories**				
Underweight, n (%)	110 (2.4)	35 (1.9)	75 (2.7)	**< 0.001^b^**
Normal weight, n (%)	1804 (39.5)	625 (34.2)	1179 (43.0)	
Overweight, n (%)	1820 (39.9)	751 (41.1)	1069 (39.0)	
Obesity, n (%)	831 (18.2)	415 (22.7)	416 (15.2)	

**Central obesity**				
No, n (%)	2373 (52.0)	830 (45.5)	1543 (56.3)	**< 0.001^b^**
Yes, n (%)	2192 (48.0)	996 (54.5)	1196 (43.7)	
**Age (years), mean ± SD**	47.48 ± 11.27	48.62 ± 11.65	46.73 ± 10.95	**< 0.001^a^**
18–29, n (%)	295 (6.5)	120 (6.6)	175 (6.4)	**< 0.001^b^**
30–39, n (%)	908 (19.9)	319 (17.5)	589 (21.5)	
40–49, n (%)	1280 (28.0)	471 (25.8)	809 (29.5)	
50–59, n (%)	1336 (29.3)	524 (28.7)	812 (29.6)	
≥60, n (%)	746 (16.3)	392 (21.5)	354 (12.9)	

**Education level**				
Primary school and below, n (%)	2307 (50.5)	599 (32.8)	1708 (62.4)	**< 0.001^b^**
Middle school, n (%)	1177 (25.8)	716 (39.2)	461 (16.8)	
High school and technical secondary school, n (%)	518 (11.3)	272 (14.9)	246 (9.0)	
College and above, n (%)	563 (12.3)	239 (13.1)	324 (11.8)	

**Living areas**				
Urban, n (%)	1811 (39.7)	721 (39.5)	1090 (39.8)	0.188^b^
Northern township, %	1657 (36.3)	642 (35.2)	1015 (37.1)	
Southern township, %	1097 (24.0)	463 (25.4)	634 (23.1)	

**Marital Status**				
Married, %	4265 (93.4)	1713 (93.8)	2552 (93.2)	0.393^b^
Unmarried/divorced/widowed, %	300 (6.6)	113 (6.2)	187 (6.8)	

**Hypertension**				
No, %	3933 (86.2)	1564 (85.7)	2369 (86.5)	0.421^b^
Yes, %	632 (13.8)	262 (14.3)	370 (13.5)	

**Diabetes**				
No, %	4370 (95.7)	1721 (94.2)	2649 (96.7)	**< 0.001^b^**
Yes, %	195 (4.3)	105 (5.8)	90 (3.3)	

**Dyslipidemia**				
No, %	2140 (46.9)	719 (39.4)	1421 (51.9)	**< 0.001^b^**
Yes, %	2425 (53.1)	1107 (60.6)	1318 (48.1)	

**Hyperuricemia**				
No, %	4248 (93.1)	1557 (85.3)	2691 (98.2)	**< 0.001^b^**
Yes, %	317 (6.9)	269 (14.7)	48 (1.8)	

*p*^a^-value was calculated by Student’s *t*-test.

*p*^b^-value was calculated by Pearson’s χ^2^ test.

Bold values indicated that the p-value was statistically significant.

### 3.2 Prevalence of overweight, obesity and central obesity

**Supplementary figure 1:**
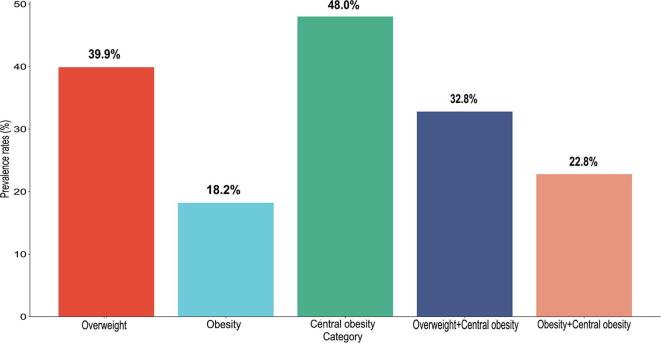


**Supplementary figure 2:**
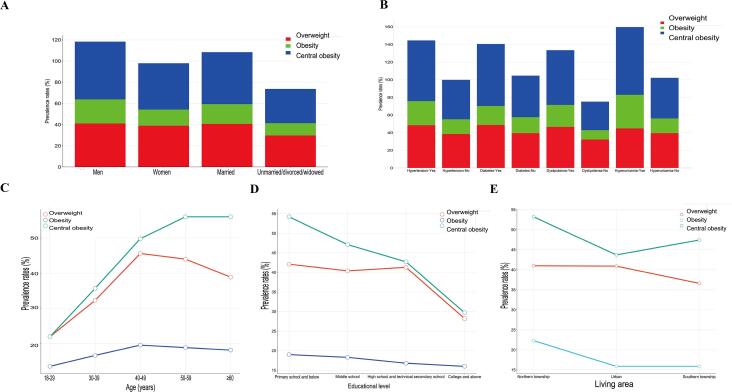


Table 2 presents the prevalence of overweight, obesity, and central obesity among adults in Shenmu, categorized by various demographic and health-related characteristics. These include sex, age, education level, residential areas (Urban, Northern township (industrial zone), Southern township (agricultural area)), marital status, and the presence of conditions such as hypertension, diabetes, dyslipidemia, and hyperuricemia. The result suggested that the overall prevalence of overweight, obesity and central obesity was 39.9 % (1820), 18.2 % (8 3 1) and 48.0 % (2192), respectively (Supplemental Fig. 1). In addition, it was worth noting that under different characteristics (Supplemental Fig. 2), there were significant differences between overweight and normal, obesity and normal and central obesity and non-central obesity (*p* < 0.05). The highest age of overweight and obesity in adults in Shenmu City was 40–49 years old, while the higher age of central obesity was over 50 years old. Similarly, Shenmu adults with primary school or below, southern township, married, and concurrent chronic diseases (including hypertension, diabetes, dyslipidemia, and hyperuricemia) have a higher prevalence.

**Table 2:** Distribution of overweight, obesity and central obesity of the studied adults in Shenmu city, Shaanxi province, 2019.

Characteristic		Total	Overweight (%)	Obesity (%)	Central obesity (%)
Total		4565	1820 (39.9)	831 (18.2)	2192 (48.0)
Sex	Men	1826	751 (41.1)	415 (22.7)	996 (54.5)
	Women	2739	1069 (39.0)	416 (15.2)	1196 (43.7)
	χ2		16.85	55.71	51.96
	*p*		**< 0.001**	**< 0.001**	**< 0.001**
Age	18–29	295	65 (22.0)	40 (13.6)	65 (22.0)
	30–39	908	293 (32.3)	152 (16.7)	324 (35.7)
	40–49	1280	584 (45.6)	251 (19.6)	637 (49.8)
	50–59	1336	588 (44.0)	252 (18.9)	748 (56.0)
	>60	746	290 (38.9)	136 (18.2)	418 (56.0)
	χ2		99.03	31.38	189.91
	*p*		**< 0.001**	**< 0.001**	**< 0.001**
Education level	Primary school and below	2307	971 (42.1)	439 (19.0)	1250 (54.2)
	Middle school	1177	476 (40.4)	215 (18.3)	554 (47.1)
	High school and technical secondary school	518	214 (41.3)	87 (16.8)	221 (42.7)
	College and above	563	159 (28.2)	90 (16.0)	167 (29.7)
	χ2		41.24	12.31	117.50
	*p*		**< 0.001**	**0.006**	**< 0.001**
Living areas	Urban	1811	740 (40.9)	288 (15.9)	791 (43.7)
	Northern township	1657	679 (41.0)	369 (22.3)	881 (53.2)
	Southern township	1097	401 (36.6)	174 (15.9)	520 (47.4)
	χ2		19.71	41.57	31.45
	*p*		**< 0.001**	**< 0.001**	**< 0.001**
Marital Status	Married	4265	1731 (40.6)	796 (18.7)	2095 (49.1)
	Unmarried/divorced/widowed	300	89 (29.7)	35 (11.7)	97 (32.3)
	χ2		15.33	13.40	31.65
	*p*		**< 0.001**	**< 0.001**	**< 0.001**
Hypertension	No	3933	1514 (38.5)	658 (16.7)	1757 (44.7)
	Yes	632	306 (48.4)	173 (27.4)	435 (68.8)
	χ2		59.49	82.70	127.29
	*p*		**< 0.001**	**< 0.001**	**< 0.001**
Diabetes	No	4370	1725 (39.5)	789 (18.1)	2055 (47.0)
	Yes	195	95 (48.7)	42 (21.5)	137 (70.3)
	χ2		9.57	5.65	40.36
	*p*		**0.002**	**0.017**	**< 0.001**
Dyslipidemia	No	2140	691 (32.3)	226 (10.6)	691 (32.3)
	Yes	2425	1129 (46.6)	605 (24.9)	1501 (61.9)
	χ2		226.59	291.59	399.23
	*p*		**< 0.001**	**< 0.001**	**< 0.001**
Hyperuricemia	No	4248	1678 (39.5)	710 (16.7)	1949 (45.9)
	Yes	317	142 (44.8)	121 (38.2)	243 (76.7)
	χ2		45.70	130.30	111.93
	*p*		**< 0.001**	**＜0.001**	**< 0.001**

*p*-value was calculated by Pearson’s χ2 test.

### 3.3 Analysis of factors related to overweight, obesity and central obesity

In order to study the risk of overweight, obesity and central obesity under different characteristics such as sex, age, education level, living areas, marital status, hypertension, diabetes, dyslipidemia, and hyperuricemia, multivariable logistic regression analysis was conducted, as shown in Table 3. Logistic regression results showed that all the above related factors were associated with overweight, obesity and central obesity. Among them, the factors that were significantly associated with reduced risk of overweight, obesity and central obesity were women (overweight: odds ratio (OR) [95 % confidence interval (95 %CI)] = 0.79 (0.69–0.91), *p* = 0.001; obesity: OR (95 % CI) = 0.55 (0.46–0.65), *p* = 0.001; central obesity: OR (95 % CI) = 0.68 (0.60–0.77), *p* = 0.001), College and above (overweight: OR (95 % CI) = 0.54 (0.44–0.66), *p* < 0.001; obesity: OR (95 % CI) = 0.54 (0.42–0.69), *p* < 0.001; central obesity: OR (95 % CI) = 0.38 (0.32–0.46), *p* < 0.001) and Unmarried/divorced/widowed (overweight: OR (95 % CI) = 0.60 (0.46–0.78), *p* < 0.001; obesity: OR (95 % CI) = 0.45 (0.30–0.65), *p* < 0.001; central obesity: OR (95 % CI) = 0.50 (0.39–0.64), *p* < 0.001). However, the factors significantly associated with an increased risk of overweight, obesity, and central obesity were age > 40 (OR > 3.37, *p* ≤ 0.001) and complications with chronic diseases such as hypertension (overweight: OR (95 % CI) = 2.11 (1.71–2.61), *p* < 0.001; obesity: OR (95 % CI) = 3.18 (2.48–4.07), *p* < 0.001; central obesity: OR (95 % CI) = 2.47 (2.06–2.98), *p* < 0.001), diabetes (overweight: OR (95 % CI) = 1.51 (1.07–2.11), *p* = 0.018; central obesity: OR (95 % CI) = 2.20 (1.6–3.02), *p* < 0.001), dyslipidemia (overweight: OR (95 % CI) = 2.57 (2.24–2.94), *p* < 0.001; obesity: OR (95 % CI) = 4.25 (3.53–5.11), *p* < 0.001; central obesity: OR (95 % CI) = 3.03 (2.68–3.44), *p* < 0.001), and hyperuricemia (overweight: OR (95 % CI) = 2.53 (1.82–3.53), *p* < 0.001; obesity: OR (95 % CI) = 4.07 (2.88–5.76), *p* < 0.001; OR (95 % CI) = 2.84 (2.16–3.72), *p* < 0.001).

**Table 3:** Association between the factors and overweight, obesity and central obesity of the studied adults by multivariate logistic regression analysis in Shenmu city, Shaanxi province, 2019.

Characteristic		Overweight		Obesity		Central obesity	
		OR (95 %CI)	*P*	OR (95 %CI)	*P*	OR (95 %CI)	*P*
Sex	Men	1		1		1	
	Women	0.79 (0.69–0.91)	**0.001**	0.55 (0.46–0.65)	**0.001**	0.68 (0.60–0.77)	**0.001**
Age	18–29	1		1		1	
	30–39	1.55 (1.06–2.26)	**0.023**	1.55 (0.98–2.46)	0.061	1.67 (1.19–2.35)	**0.003**
	40–49	3.73 (2.33–5.97)	**< 0.001**	3.50 (1.99–6.18)	**< 0.001**	3.78 (2.48–5.75)	**< 0.001**
	50–59	3.96 (2.27–6.94)	**< 0.001**	4.16 (2.11–8.20)	**< 0.001**	5.95 (3.60–9.82)	**< 0.001**
	>60	3.37 (1.74–6.52)	**< 0.001**	4.13 (1.86–9.18)	**0.001**	6.85 (3.80–12.35)	**< 0.001**
Education level	Primary school and below	1		1		1	
	Middle school	0.83 (0.71–0.97)	**0.019**	0.70 (0.57–0.85)	**< 0.001**	0.60 (0.53–0.70)	**< 0.001**
	High school and technical secondary school	0.90 (0.73–1.12)	0.340	0.71 (0.54–0.94)	**0.015**	0.54 (0.44–0.65)	**< 0.001**
	College and above	0.54 (0.44–0.66)	**< 0.001**	0.54 (0.42–0.69)	**< 0.001**	0.38 (0.32–0.46)	**< 0.001**
Living areas	Urban	1		1		1	
	Northern township	1.10 (0.95–1.28)	0.213	1.49 (1.24–1.80)	**< 0.001**	1.23 (1.07–1.40)	**0.003**
	Southern township	0.74 (0.63–0.87)	**< 0.001**	0.78 (0.63–0.97)	**0.025**	0.96 (0.82–1.11)	0.541
Marital Status	Married	1		1		1	
	Unmarried/divorced/widowed	0.60 (0.46–0.78)	**< 0.001**	0.45 (0.30–0.65)	**< 0.001**	0.50 (0.39–0.64)	**< 0.001**
Hypertension	No	1		1		1	
	Yes	2.11 (1.71–2.61)	**< 0.001**	3.18 (2.48–4.07)	**< 0.001**	2.47 (2.06–2.98)	**< 0.001**
Diabetes	No	1		1		1	
	Yes	1.51 (1.07–2.11)	**0.018**	1.48 (0.98–2.25)	0.065	2.20 (1.6–3.02)	**< 0.001**
Dyslipidemia	No	1		1		1	
	Yes	2.57 (2.24–2.94)	**< 0.001**	4.25 (3.53–5.11)	**< 0.001**	3.03 (2.68–3.44)	**< 0.001**
Hyperuricemia	No	1		1		1	
	Yes	2.53 (1.82–3.53)	**< 0.001**	4.07 (2.88–5.76)	**< 0.001**	2.84 (2.16–3.72)	**< 0.001**

*p*-value was calculated by logistic regression analysis with adjustments for age and gender.

Bold values indicated that the *p*-value was statistically significant.

### 3.4 Prevalence of overweight/obesity combined with central obesity

**Supplementary figure 3:**
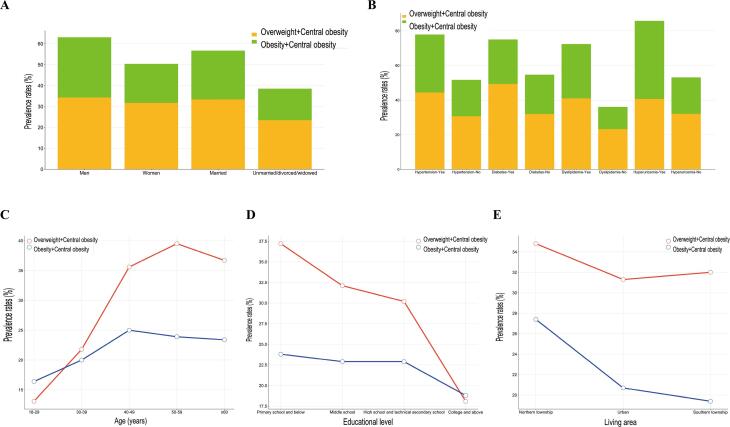


Table 4 presents the prevalence rates of overweight combined with central obesity and obesity combined with central obesity among adults in Shenmu, categorized by various characteristics such as sex, age, education level, living areas, marital status, hypertension, diabetes, dyslipidemia, and hyperuricemia. The study found that the prevalence of overweight combined with central obesity was 32.8 % (1,132 participants) and obesity combined with central obesity was 22.8 % (789 participants), as shown in Supplemental Fig. 1 Notably, there were significant differences in the prevalence of overweight combined with central obesity and obesity combined with central obesity across different demographic and health categories, as illustrated in Supplemental Fig. 3 (*p* < 0.05). The highest prevalence of overweight combined with central obesity was observed in participants aged 50–59 years, whereas the highest prevalence of obesity combined with central obesity was found in the 40–49 age group in Shenmu City. Furthermore, the groups with a high prevalence of overweight or obesity combined with central obesity primarily included men, those with an education level of primary school or below, residents of the northern township, married individuals, and those with conditions like hypertension, diabetes, dyslipidemia, and hyperuricemia.

**Table 4:** Distribution of overweight/obesity combined with central obesity of the studied adults in Shenmu city, Shaanxi province, 2019.

Characteristic		Total	Overweight＋Central obesity (%)	Obesity＋Central obesity (%)
Total		3455	1132 (32.8)	789 (22.8)
Sex	Men	1410	484 (34.3)	406 (28.8)
	Women	2045	648 (31.7)	383 (18.7)
	χ2		21.77	67.01
	*p*		**< 0.001**	**< 0.001**
Age	18–29	214	28 (13.1)	35 (16.4)
	30–39	696	152 (21.8)	139 (20.0)
	40–49	961	342 (35.6)	240 (25.0)
	50–59	1023	404 (39.5)	244 (23.9)
	≥60	561	206 (36.7)	131 (23.4)
	χ2		145.87	52.15
	*p*		**< 0.001**	**< 0.001**
Education level	Primary school and below	1775	660 (37.2)	422 (23.8)
	Middle school	881	283 (32.1)	202 (22.9)
	High school and technical secondary school	367	111 (30.2)	84 (22.9)
	College and above	432	78 (18.1)	81 (18.8)
	χ2		82.35	27.04
	*p*		**< 0.001**	**< 0.001**
Living areas	Urban	1345	421 (31.3)	278 (20.7)
	Northern township	1269	442 (34.8)	348 (27.4)
	Southern township	841	269 (32.0)	163 (19.4)
	χ2		17.63	38.10
	*p*		**< 0.001**	**< 0.001**
Marital Status	Married	3242	1082 (33.4)	757 (23.3)
	Unmarried/divorced/widowed	213	50 (23.5)	32 (15.0)
	χ2		17.50	16.06
	*p*		**< 0.001**	**< 0.001**
Hypertension	No	2944	905 (30.7)	618 (21.0)
	Yes	511	227 (44.4)	171 (33.5)
	χ2		94.22	99.38
	*p*		**< 0.001**	**0.001**
Diabetes	No	3295	1053 (32)	748 (22.7)
	Yes	160	79 (49.4)	41 (25.6)
	χ2		29.19	10.38
	*p*		**< 0.001**	**< 0.001**
Dyslipidemia	No	1599	371 (23.2)	206 (12.9)
	Yes	1856	761 (41.0)	583 (31.4)
	χ2		299.13	343.21
	*p*		**< 0.001**	**< 0.001**
Hyperuricemia	No	3187	1023 (32.1)	668 (21.0)
	Yes	268	109 (40.7)	121 (45.1)
	χ2		63.95	135.11
	*p*		**< 0.001**	**< 0.001**

*p*-value was calculated by Pearson’s χ2 test.

### 3.5 Analysis of related factors of overweight/obesity combined with central obesity

In order to study the risk of overweight combined with central obesity and obesity combined with central obesity under different characteristics such as sex, age, education level, living areas, marital status, hypertension, diabetes, dyslipidemia and hyperuricemia, multivariable logistic regression analysis was conducted, as shown in Table 5. Logistic regression showed that the above related factors were related to overweight combined with central obesity and obesity combined with central obesity. Among them, the factors significantly associated with reduced risk of overweight/obesity combined with central obesity were women (overweight with central obesity: OR (95 %CI) = 0.76 (0.65–0.90), *p* = 0.001; obesity with central obesity: OR (95 %CI) = 0.51 (0.43–0.61), *p* < 0.001), Middle school and above (OR < 0.74, *p* ≤ 0.038), and unmarried/divorced/widowed (overweight with central obesity: OR (95 %CI) = 0.65 (0.46–0.92), *p* = 0.016; obesity with central obesity: OR (95 %CI) = 0.53 (0.35–0.80), *p* = 0.002). However, the factors significantly associated with increased risk of overweight combined with central obesity and obesity combined with central obesity were age > 40 (OR > 2.81, *p* < 0.001), northern township (overweight with central obesity: OR (95 %CI) = 1.36 (1.13–1.63), *p* = 0.001; obesity with central obesity: OR (95 %CI) = 1.65 (1.35–2.02), *p* < 0.001) and complications of chronic diseases including hypertension (overweight with central obesity: OR (95 %CI) = 2.40 (1.87–3.08), *p* < 0.001; obesity with central obesity: OR (95 %CI) = 3.22 (2.45–4.23), *p* < 0.001), diabetes (overweight with central obesity: OR (95 %CI) = 2.05 (1.38–3.04), *p* < 0.001; obesity with central obesity: OR (95 %CI) = 1.58 (1.00–2.50), *p* = 0.048), dyslipidemia (overweight with central obesity: OR (95 %CI) = 3.62 (3.07–4.28), *p* < 0.001; obesity with central obesity: OR (95 %CI) = 5.26 (4.33–6.39), *p* < 0.001), and hyperuricemia (overweight with central obesity: OR (95 %CI) = 4.37 (2.94–6.48), *p* < 0.001; obesity with central obesity: OR (95 %CI) = 6.34 (4.28–9.39), *p* < 0.001).

**Table 5:** Association of the factors with overweight/obesity combined with central obesity of the studied adults by multivariate logistic regression analysis in Shenmu city, Shaanxi province, 2019.

Characteristic		Overweight combined with central obesity		Obesity combined with central obesity	
		OR (95 %CI)	*P*	OR (95 %CI)	*P*
Sex	Men	1		1	
	Women	0.76 (0.65–0.90)	**0.001**	0.51 (0.43–0.61)	**< 0.001**
Age	18–29	1		1	
	30–39	2.08 (1.33–3.25)	**0.001**	1.55 (1.02–2.36)	**0.041**
	40–49	4.91 (3.20–7.55)	**< 0.001**	2.81 (1.87–4.22)	**< 0.001**
	50–59	5.81 (3.78–8.90)	**< 0.001**	2.78 (1.85–4.17)	**< 0.001**
	>60	4.74 (3.03–7.40)	**< 0.001**	2.22 (1.44–3.41)	**< 0.001**
Education level	Primary school and below	1		1	
	Middle school	0.74 (0.60–0.90)	**0.003**	0.68 (0.54–0.85)	**0.001**
	High school and technical secondary school	0.69 (0.52–0.90)	**0.007**	0.73 (0.54–0.98)	**0.038**
	College and above	0.46 (0.34–0.63)	**< 0.001**	0.52 (0.38–0.72)	**< 0.001**
Living areas	Urban	1		1	
	Northern township	1.36 (1.13–1.63)	**0.001**	1.65 (1.35–2.02)	**< 0.001**
	Southern township	1.00 (0.82–1.23)	0.988	0.89 (0.70–1.12)	0.316
Marital Status	Married	1		1	
	Unmarried/divorced/widowed	0.65 (0.46–0.92)	**0.016**	0.53 (0.35–0.80)	**0.002**
Hypertension	No	1		1	
	Yes	2.40 (1.87–3.08)	**< 0.001**	3.22 (2.45–4.23)	**< 0.001**
Diabetes	No	1		1	
	Yes	2.05 (1.38–3.04)	**< 0.001**	1.58 (1.00–2.50)	**0.048**
Dyslipidemia	No	1		1	
	Yes	3.62 (3.07–4.28)	**< 0.001**	5.26 (4.33–6.39)	**< 0.001**
Hyperuricemia	No	1		1	
	Yes	4.37 (2.94–6.48)	**< 0.001**	6.34 (4.28–9.39)	**< 0.001**

*p*-value was calculated by logistic regression analysis with adjustments for age and gender.

Bold values indicated that the *p*-value was statistically significant.

## 4 Discussion

Overweight, obesity, and central obesity are increasingly recognized as major global public health issues. It is projected that by 2030, approximately 1.35 billion and 573 million adults in China will be overweight and obese, respectively (Kelly et al., 2008). Currently, health problems and medical costs directly or indirectly associated with obesity are imposing a substantial economic burden on nations and societies. For instance, in 2020, the total cost of obesity in Italy was estimated at €13.34 billion, with cardiovascular diseases (CVD) being the most significant cost factor, followed by diabetes, cancer, and bariatric surgery (d'Errico et al., 2022). Recent research suggests that by 2030, medical expenses related to overweight/obesity in China could reach 418 billion yuan, accounting for about 21.5 % of the country’s total medical expenditures (Wang et al., 2021b). This underscores the significant medical and economic impact of obesity on individuals, countries, and societies.

This study analyzed the prevalence of overweight, obesity, central obesity, and their combinations among adult residents of Shenmu, Shaanxi Province. The findings revealed prevalence rates of 39.9 % for overweight, 18.2 % for obesity, 48 % for central obesity, 32.8 % for overweight combined with central obesity, and 22.8 % for obesity combined with central obesity. Notably, the prevalence of central obesity was higher than that of other categories, suggesting that, compared to systemic obesity, central obesity is more prevalent among adults in Shenmu City. Recent studies have linked abdominal obesity with insulin resistance and a heightened risk of metabolic syndrome and cardiovascular diseases, more so than overall obesity (Goh and Hart, 2018). Other research indicates that central obesity is a more accurate predictor of cardiovascular disease risk than systemic obesity and is considered a significant risk factor for cardiovascular and metabolic diseases (Piché et al., 2018). Specifically, a study in Shaanxi, China, found that normal weight individuals with central obesity had an increased risk of cardiovascular diseases, such as hypertension and dyslipidemia, regardless of sex (Feng et al., 2021). Therefore, central obesity warrants special attention from relevant authorities.

The prevalence and risk factors of overweight, obesity, and central obesity among adult residents of Shenmu City appear to be influenced by various factors. Regarding gender, the prevalence of overweight, obesity, and central obesity is generally higher in men than in women. This finding aligns with the research by Junjie Hua et al. (Hua et al., 2020, Wang et al., 2021a), The prevalence and risk factors of overweight, obesity, and central obesity among adult residents of Shenmu City appear to be influenced by various factors. Regarding gender, the prevalence of overweight, obesity, and central obesity is generally higher in men than in women. This finding aligns with the research by Junjie Hua et al. (Liu et al., 2021) and may be linked to the increased family responsibilities often associated with married life.

In addition to the aforementioned factors, hypertension, diabetes, dyslipidemia, and hyperuricemia are also intricately linked to overweight, obesity, and central obesity. Studies indicate that Shenmu residents with these conditions are more susceptible to overweight, obesity, central obesity, and a combination of overweight/obesity with central obesity. These findings align with related research (Movahed et al., 2016, Bragg et al., 2018, Lee et al., 2022, Yu et al., 2016), underscoring the importance of managing hypertension, diabetes, dyslipidemia, and hyperuricemia in the prevention of overweight, obesity, and central obesity. However, as this study is cross-sectional in nature, the causal relationships between these health conditions and various forms of obesity warrant further investigation through cohort studies.

In conclusion, the prevalence of overweight, obesity, and central obesity among adult residents in Shenmu City is high, with multivariable influencing factors, including gender, age, education level, living areas, economic status, marital status, and chronic metabolic diseases. Fortunately, these conditions are preventable and controllable. This necessitates awareness from relevant authorities, who should engage in health promotion activities aligned with the 'Healthy China 2030′ initiative, develop effective prevention and control strategies, and work towards improving the health of the population at large.

## 5 Conclusions

Recent research indicates that adult residents in Shenmu City exhibit high prevalence rates of overweight, obesity, central obesity, and combinations of overweight or obesity with central obesity. Particularly noteworthy is the prevalence of central obesity, which significantly increases the risk of cardiovascular diseases and chronic metabolic disorders among the city's population. This study offers valuable insights and serves as a reference for developing effective intervention measures and prevention strategies to combat obesity.

## 6 Ethics statement

This study was approved by the Ethics Committee of Shenmu Hospital (NO.sm004), and all subjects signed informed consent.

## Funding

This work was supported by 10.13039/501100007128Natural Science Foundation of Shaanxi Province (2021SF-075), Science and Technology Plan Project of Yulin City (YF-2020-191) and Shenmu Municipal Government Scientific Research Project (2019) No. 5.

## Author contributions

Yonglin Liu and Shengli Sun contributed to the conception and design of the work; Mingxia Liu and Chunjiao Jia acquired the data; Yaoda Hu, Juan Liu, Lizhen Liu and Haiying Wang conducted the statistical analysis. All authors provided substantial scientific input in interpreting the results, drafting and or reviewing the manuscript.

## CRediT authorship contribution statement

**Mingxia Liu:** Writing – review & editing, Writing – original draft. **Chunjiao Jia:** Writing – review & editing, Writing – original draft. **Yaoda Hu:** Methodology, Formal analysis, Data curation. **Juan Liu:** Software, Methodology. **Lizhen Liu:** Validation, Software. **Shengli Sun:** Validation. **Haiying Wang:** Validation. **Yonglin Liu:** Writing – review & editing, Conceptualization.

## Declaration of competing interest

The authors declare that they have no known competing financial interests or personal relationships that could have appeared to influence the work reported in this paper.

## Data Availability

Due to concerns for participant privacy, data are available only upon reasonable request to the corresponding author.
